# Combining RNA-seq and proteomic profiling to identify seminal fluid proteins in the migratory grasshopper *Melanoplus sanguinipes* (F)

**DOI:** 10.1186/s12864-015-2327-1

**Published:** 2015-12-22

**Authors:** Martha L. Bonilla, Christopher Todd, Martin Erlandson, Jose Andres

**Affiliations:** Facultad de Ciencias Agropecuarias, Universidad Nacional de Colombia, A.237. Palmira, Valle del Cauca, Colombia; Department of Biology, University of Saskatchewan, 112 Science Pl., Saskatoon, SK S7N-5E2 Canada; Saskatoon Research Centre, Agriculture and Agri-Food Canada, 107 Science Pl., Saskatoon, SK S7N-0X2 Canada

**Keywords:** Transcriptome, Proteomics, Next generation sequencing, Agricultural pest, Male reproductive genes, *Orthoptera*

## Abstract

**Background:**

Seminal fluid proteins control many aspects of fertilization and in turn, they play a key role in post-mating sexual selection and possibly reproductive isolation. Because effective proteome profiling relies on the availability of high-quality DNA reference databases, our knowledge of these proteins is still largely limited to model organisms with ample genetic resources. New advances in sequencing technology allow for the rapid characterization of transcriptomes at low cost. By combining high throughput RNA-seq and shotgun proteomic profiling, we have characterized the seminal fluid proteins secreted by the primary male accessory gland of the migratory grasshopper (*Melanoplus sanguinipes*), one of the main agricultural pests in central North America.

**Results:**

Using RNA sequencing, we characterized the transcripts of ~ 8,100 genes expressed in the long hyaline tubules (LHT) of the accessory glands. Proteomic profiling identified 353 proteins expressed in the long hyaline tubules (LHT). Of special interest are seminal fluid proteins (SFPs), such as *EJAC-SP*, *ACE* and prostaglandin synthetases, which are known to regulate female oviposition in insects.

**Conclusions:**

Our study provides new insights into the proteomic components of male ejaculate in Orthopterans, and highlights several important patterns. First, the presence of proteins that lack predicted classical secretory tags in accessory gland proteomes is common in male accessory glands. Second, the products of a few highly expressed genes dominate the accessory gland secretions. Third, accessory gland transcriptomes are enriched for novel transcripts. Fourth, there is conservation of SFPs’ functional classes across distantly related taxonomic groups with very different life histories, mating systems and sperm transferring mechanisms. The identified SFPs may serve as targets of future efforts to develop species- specific genetic control strategies.

**Electronic supplementary material:**

The online version of this article (doi:10.1186/s12864-015-2327-1) contains supplementary material, which is available to authorized users.

## Background

Insect seminal fluid proteins (SFPs), produced by the male accessory gland (AG), not only contribute to spermatophore formation (the capsules containing the ejaculate) but they also influence individual fitness by modifying different aspects of the females’ reproductive physiology and behavior [1–10]. For this reason, SFPs have been the focus of an increasing number of evolutionary studies on sexual conflict, post-mating sexual selection, and speciation [4, 5, 11–18]. A less explored aspect of the relationship between SFPs and fitness is the potential for the development of new pest control strategies aimed at disrupting the reproductive cycle of insect vectors and pests [19]. In insects, many SFP-encoding genes are highly divergent and show signs of positive selection [20–23]. Therefore, these loci are potential targets for the future development of species-specific, gene silencing biopesticides based on specific genetic control strategies.

Increasingly sophisticated molecular tools and the ability to generate massive amounts of genomics and proteomics data makes it possible to identify the proteins that are transferred to females during copulation. During the last decade, we have witnessed a rapid increase in the number of studies dissecting the structure and function of SFPs in a broad range of insects [6, 9, 19, 24–44]. However, these studies have only characterized the protein composition of the seminal fluid in a handful of taxonomic groups and the SFPs of most of the disease vectors and agricultural pest insects have yet to be identified.

Orthopterans are a large order of insects with more than 20,000 species, including a number of economically significant pests [9]. Only five species of crickets, however, (4 *Grylloidea* and 1 *Tettigonioidea*) have been studied thus far [14, 16, 17, 25, 42, 45–47]. Swarming and migratory grasshoppers (Family *Acrididae*) have been long recognized as crop pests, since the origins of cultivation ~10,000 years ago. In western North America, grasshopper feeding results in an estimated annual loss of $1.25 billion per year [48]. Here, as a first step to identify the repertoire of SFPs in economically relevant species of grasshoppers, we aim to characterize the protein secretome of the long hyaline tubules in the migratory grasshopper (*Melanoplus sanguinipes*), one of the main pest species feeding on cereal and crucifer crops in central North America [47].

*M. sanguinipes* males are promiscuous and mate frequently, transferring up to fourteen small tubular spermatophores with each mating. During copulation, male spermatophores penetrate a short distance into the female’s spermathecal duct where the seminal fluid is discharged before they are withdrawn and become lodged between the female’s genital valvulae [49]. Both spermatophore building proteins and SPFs are produced in the complex group of multi-paired male accessory glands. These include one pair of long hyaline tubules, four pairs of white glands, ten pairs of short hyaline tubules and one pair of seminal vesicles [50]. Each of these type of glands secretes different proteins at specific times as the male develops [51]. Of special relevance are the long hyaline tubules (LHT) that produce large amounts of a partially identified protein (Oviposition Stimulating Protein, OSP) that is transferred from males to females during copulation and stimulates oviposition [50–53]. OSP is the major component that it is discharged with the spermatophores. Other LHT-secreted proteins seem to contribute to the formation and correct uncoiling of spermatophores [54]. Using a combination of next- generation RNA sequencing and proteomic analysis, we have identified and characterized the SFPs expressed in this gland. These include not only OSP but also novel transcripts and protein classes previously described in insects with very different mating and seminal fluid transferring systems.

## Methods

### Sampling of insects and tissue dissection

**Fig. 1:**
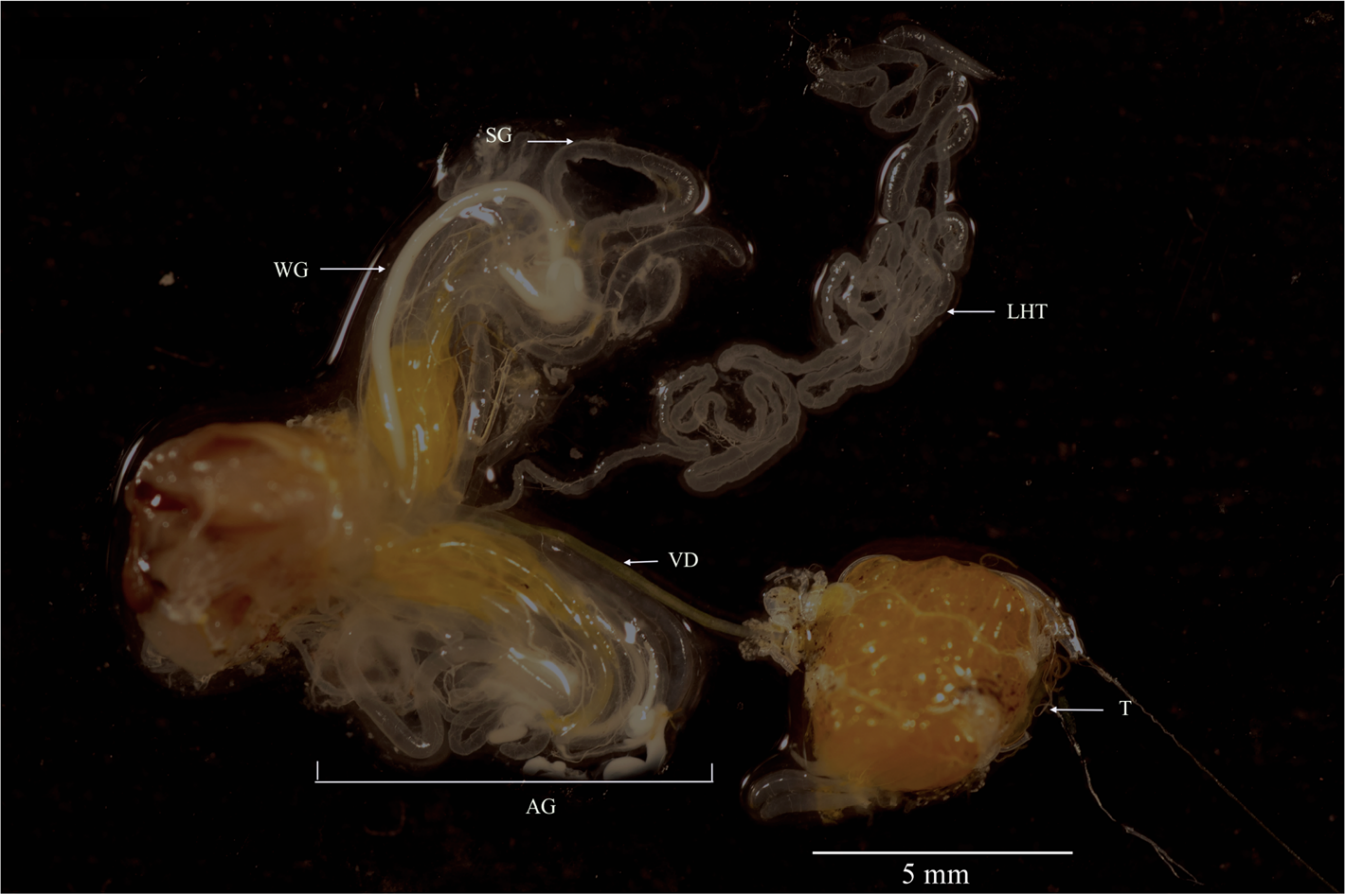
Male reproductive system of *M. sanguinipes*. LHT, Long hyaline tubules; T, testes; VD, vas deferens; WG, white glands SW, Short hyaline glands, AG, accessory gland complex

Migratory grasshopper males are sexually mature a week after their last moult [55]. All experiments were conducted using 10-day-old males from the nondiapause colony of *M. sanguinipes,* maintained at Agriculture and Agri-Food Canada’s Saskatoon Research Centre (Saskatoon, SK). Rearing methods are those described in [56]. All male accessory glands were dissected in Ringer’s buffer and the LHT was carefully isolated (Fig. 1).

### cDNA library construction and assembly

Total RNA was extracted from the LHT of 9 mated and 9 virgins’ adult males using TRIZOL (Invitrogen, Carlsbad, CA). Immediately after, the mRNA fraction was isolated using a Poly (A) Purist kit (Ambion), and checked its quality using an RNA 6000 Nano Kit and a Bioanalyzer 2100 (Agilent). Then the mRNA sample was fragmented and size selected (300 bp-1Kb) using the high temperature Zn^2+^ method and a cDNA Rapid Library Prep kit (Roche). Size fractionated samples were then sequenced at the National Research Council Plant Biotechnology Institute Canada (NRC-Saskatoon) using 454 GS-FLX platform and Titanium™ chemistry (Roche). Reads were *de novo* assembled using CLC Genomics Workbench software v 5.0 (similarity = 0.90, length fraction = 0.5, Mismatch cost = 2, indel cost = 3), into contigs with a minimum size cutoff of 200 bp.

### Transcriptome annotation and homology-based functional analyses

Assembled transcripts were submitted for homology, annotation searching and Gene Ontology (GO) categorization, using the online version of Blast2go program (www.blast2go.com) [57]. Homology searches were done against non-redundant databases of the National Center for Biotechnology information (NCBI) using BLASTx algorithm. The annotations step was generated retrieving the keywords from the BLASTx descriptions. Gene Ontology categorizations of the functional annotations were carried out on the top BLASTx hits (1e-5 cutoff).

Search of putative open reading frames (ORFs) of at least 30 codons (90 bp) was done using the function Getorf from EMBOSS software [58]. The presence of conserved protein domains was tested using the online version of the program Pfam (http://pfam.sanger.ac.uk/) [59].

### Identification of SFPs through mass-spectometry

In insect species in which males transfer a single external spermatophore, SFPs can be identified using mass-spectrometry by comparing the spectra of peptides directly isolated from the spermatophore to that of a species-specific protein database (e.g. [40]). However this is not always possible. For example, in species such as the migratory grasshopper (in which males sequentially transfer up to fourteen spermatophores during a single copulation) it is impossible to collect all spermatophores without disrupting the mating pairs [49, 60]. In such cases one can possibly use isotopic labeling of female proteins to identify male transferred SFPs [19, 61–63]. However, this is not always possible. An alternative strategy in such cases is to identify the peptides secreted by the male accessory glands of interest. Although this approach has limitations and cannot distinguish between spermatophore-building proteins and those transferred to the females, it has been successfully applied to identify SFPs in other insect species [34, 40, 42, 64]. Thus, a single pool of ten LHT excised from individual males was prepared as described above. These glands were transferred to ice-cold insect Ringer buffer (153 mM NaCl, 2.68 mM KCl, 1.36 mM CaCl_2_), and gently centrifuged at 1,000 g for 1 minute to separate the secretion of the gland contents from the tissue. The resulting supernatant was stored at −80 °C. We considered these supernatant samples as “SFP-enriched” as they contained not only secreted proteins, but also likely LHT tissue. In-gel digestion, tryptic peptide extractions and Nano liquid chromatography with tandem mass spectrometry (LC-MS/MS) were conducted by Genome BC Proteomics Centre at the University of Victoria, Victoria, British Columbia, Canada. Proteins from the viscous secretions were separated by electrophoresis on a one-dimensional 5–15 % gradient SDS polyacrylamide gel (Additional file 1: Figure S1) and visualized using Simply-Blue SafeStain (Invitrogen, Carlsbad, CA). The entire running lane of the gel was then sliced into 16 different equal size fractions. Each fraction was then transferred to a Genomics Solutions Progest perforated digestion tray, de-stained (50/45/5 (v/v) methanol/water/acetic acid) prior to reduction with 10 mM dithiothreitol (Sigma) and alkylation with100 mM iodoacetamide (Sigma), and digested for 5 h at 37 °C using sequencing grade porcine trypsin solution (20 ng/μL, Promega, Madison, WI) at an enzyme: protein ratio of 1:50. Each sample was then lyophilized following acid extraction (50/40/10 acetonitrile/water/formic acid) and stored at −80 °C prior to mass spectrometry analysis.

The peptide mixtures were separated by on-line reversed phase chromatography using a Thermo Scientific EASY-nLC II system with a reversed-phase pre-column Magic C-18AQ (100μm I.D., 3 cm length, 5μm, 100Å, Michrom BioResources Inc, Auburn, CA) and a reversed phase nano-analytical column Magic C-18AQ (75 μm I.D., 15 cm length, 5μm, 100Å, Michrom BioResources Inc, Auburn, CA). The chromatography system was coupled to an LTQ Orbitrap Velos mass spectrometer equipped with a Nanospray Flex source (Thermo Fisher Scientific). MS/MS spectra were analyzed with Proteome Discoverer 1.4.0.228 software suite (Thermo Scientific). Peak lists generated of the Collision Induced Dissociation (CID) spectra were submitted to an in-house Mascot 2.4 server to identify proteins by searching against two protein databases (Uniprot tremble and Swissprot) and a six-reading frame translation of the LHT-cDNA library previously generated, with 260,330 potential open reading frames (ORFs) to confirm correct existing transcripts by peptide spectrum matches. The default search settings used for protein identification were: MS/MS accuracies were set to < 0.6 Da, and two missed cleavages for full trypsin with fixed modifications Carbamidomethyl (C); variable modifications: deamidation (N, Q); oxidation (M) and propionamide (C). A protein was positively identified if the ion score value of at least two different peptides exceeded the significance threshold (p < 0.05). Proteins matching only one peptide (*p* < 0.05) were only positively identified if the ion score value of the matching peptide was at least double the significance threshold [65]. The relative quantitation of identified proteins was then estimated using the exponentially modified protein abundance index (emPAI, Additional file 2: Table S1) [66]. For each identified protein a single emPAI value was obtained by adding the data from all gel slices.

To identify which proteins are part of the LHT-secretome first the program SignalP 4.0 (http:// www.cbs.dtu.dk/services/SignalP) [67] was used to predict secreted proteins based on the presence and location of a signal peptide. Then, a neuronal network analysis (SecretomeP 2.0 (http://www.cbs.dtu.dk/services/Secretome P/) was implemented to further identify proteins putatively secreted by non-classical secretory pathways (*i.e.* Scoring function > 0.5) [68]. All putatively secreted proteins were used as queries in local BLASTP searches against a combined database including annotated SFPs from *Drosophila melanogaster* [29], *Aedes aegypti* [65], *Heliconius* butterflies [32], *Tribolium casteanum* [24] and *Allonemobius* and *Gryllus* crickets [14, 40, 46, 69]*.* Following [35], Pairs of sequences that had reciprocal best Blast hits (RBBHs) with e-values < 1 × 10^−3^, identities ≥ 30 % and bit score ≥100 were considered putative orthologous.

### Phylogenetic analyses

The secreted peptide known as OSP, is the most abundant protein secreted by the LHT and the only oviposition factor identified in *M. sanguinipes*. To gain further insight on its function, we carried out a multiple alignment of the published amino acid sequences of insect’s takeout/juvenile hormone-binding proteins (JHBPs) including several SFPs that have been tentatively included in this superfamily [24, 48, 54, 67, 70–74]. Phylogenetic analyses were conducted using Neighbor-joining methods and the Jones–Taylor–Thornton (JTT [75]) substitution matrix, as implemented in PAUP* b1.0 [76]. The confidence of the tree topology was assessed by a bootstrap (*n* = 1,000 replicates).

### Expression patterns of putative SFPs

For the subset of putative *M. sanguinipes* SFPs’ that have a canonical signal peptide and/or showed significant orthology with SFPs described in other insects we investigated the expression patterns analysis using a qualitative reverse transcription polymerase chain reaction (RT-PCR) [31, 32, 40]. Total RNA was extracted from adult virgin individuals (7–10 days old; *n* = 5 individuals/sex) and five different tissues: male accessory gland (AG), testes (T), male head and leg (MHL) female spermatheca and oviduct (RTF), and female head and leg (FHL). Abdominal and thorax tissues were not included in this analysis to avoid potential contamination with reproductive tissues. For each tissue, cDNA was synthesized from 1 ug of total RNA of the pooled individual samples using a Quantitect Reverse Transcription kit (Qiagen). Consequently, it was not possible to capture individual variation in the expression levels, and further studies are needed to address this point. All RT-PCRs were performed using the touchdown PCR protocol (see Additional file 3: Table S2 for PCR and primers details).

## Results

### cDNA library annotation and characterization

Sequencing using 454 GS FLX titanium technology generated 259.565 high quality reads (92,250,778 bases) that assembled into 82 singletons and 8,056 contigs. Read length of transcripts ranged from 62 to 6,208 bp with average contig length of 649 bp. We did not find a significant correlation between transcripts length and number of reads (*r* = 0.08, *p* > 0.05; *n* = 8,138).

Analysis of the expressed sequence tags (ESTs) frequency spectrum revealed that most of the transcripts (~66 %) occurred as either singletons or contigs that included only a small number of reads (*n* ≤ 5). Only a small group (*n* = 36) was represented by a high (*n* > 500) number of reads. Nucleotide-based Blast analyses (BLASTx) revealed that ~60 % of the contigs show significant similarities with either annotated gene products and/or known protein domains (E-value ≤ 10^−5^), Only a small fraction (4.4 %) showed significant homology to the same annotated transcript.

Gene Ontology (GO) assignments were used to classify the functions of the predicted genes based on contigs with significant BLASTx (E- value ≤ 10^−5^). Contigs were assigned to 23 biological processes, 9 cell components and 14 molecular functions based on GO level II (Additional file 4: Figure S2A-C). Some contigs were associated with multiple GO annotations because a single sequence may be annotated in any or all categories within a single category, giving more “GO” annotations than sequences annotated [33]. Within the biological Processes, 42 % of the contigs were assigned to metabolic and basic cellular process. Remaining contigs were involved in a broad range of biological process such as: biological regulation (10 %), developmental process (7 %), signal transduction (5 %), localization (7 %), reproductive process (2 %), cellular adhesion (1 %), response to stress (7 %) and immune response (1 %).

### Identification of putative SFPs

**Fig. 2:**
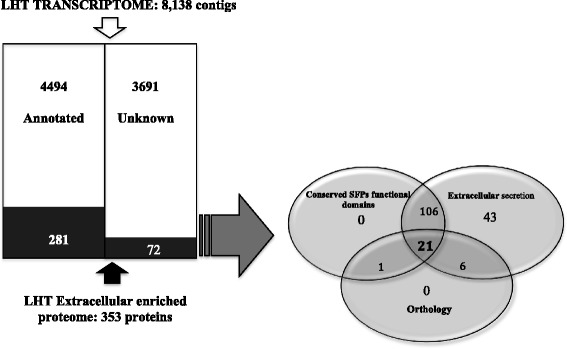
Flowchart of the strategy followed for the identification of putative SFPs

**Fig. 3:**
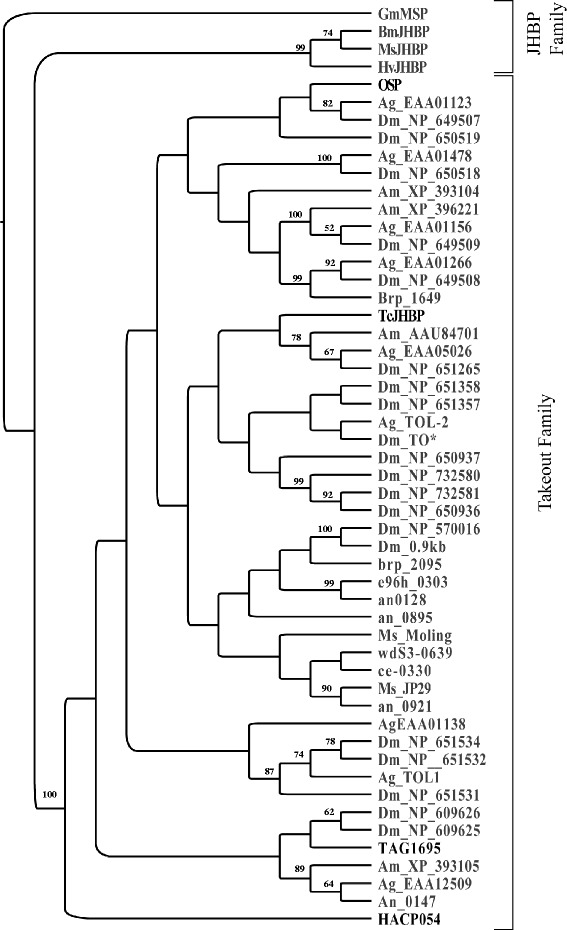
Phylogenetic tree based on published amino acid sequences of takeout/JHPB family proteins. Phylogenetic analyses were conducted using Neighbor-joining methods and the Jones–Taylor–Thornton substitution matrix (JTT, Jones *et al*. 1992). The numbers on each branch represent bootstrap values (*n* = 1,000). Known SFPs with predicted JHBP binding domain are shown in bold. Species codes and number of individual sequences included in the analysis *Drosophila melanogaster*, Dm [112, 113]; *Anopheles gambiae*,Ag [72]; *Manduca sexta*, Ms [114, 115]; *Apis mellifera*, Am [80]; *Bombyx mori*, BmJHBP, ce- 0303, brp_2095, e96h_0303, an_128, an_0895, wdS3-0639, an_0921, an_0147 [75]; *Heliothis virescens*, Hv [116]; *Galleria mellonella*, Gm [117]; *Tribolium castanenum*, Tc [24]; *Ceratitis capitata,* TAG1695 [73]; *Heliconius melpomene,* HAC054 [32]

Identification of candidate SFPs was based on combination of both, transcriptome and proteomic analyses (Fig. 2). Using annotation analyses we initially identified 4,497 LHT-expressed genes (Additional file 5: Table S3). We further identified LHT seminal proteins using a combination of mass spectrometry and bioinformatics as follows: First, we compared the peptide sequences from tryptic digests of our long hyaline tubule SFP-enriched fractions with peptide sequences generated *in silico* from a translation of the LHT transcriptome. This search resulted in the identification of 353 gene products (Additional file 2: Table S1), of which 28 % (*n* = 99) were only matched by a single-peptide. Most of these products correspond to a broad range of diverse protein families, including metabolic and structural proteins, as expected if the protein sample contained traces of LHT tissue. However, as expected if most of these proteins indeed represent SFP both the average number of reads and exponentially modified protein abundance values (emPAI) were higher in this group than in the rest of the transcriptome (N _PUTATIVE SFPs_ = 744.9 +/− 429.3, N _TRANSCRIPTOME_ = 22.2 +/− 5.44, permutation test p <0.001: emPAI _PUTATIVE SFPs_ = 2207.7 +/− 2192.8, emPAI _TRANSCRIPTOME_ = 3.6 +/− 0.65, permutation test p <0.001). Also, for this group of proteins we found a positive correlation between gene and protein expression levels (Spearman-Rho = 0.29, *p* = 0.01). In an attempt to identify *bona fide* SFPs we first selected those transcripts potentially encoding extracellular, secreted, proteins (*i.e*. contigs with a predicted signal peptide or secreted via the non-classical pathway). As expected, such analyses revealed a significant number of gene products (176/353) that are potentially transferred to the female during mating. Homology-based functional analyses revealed that the most abundant LHT-protein (EmPAI value 151323.39) is approximately 460x more abundant than the second- most abundant protein. This transcript contains a single putative juvenile hormone (JH) binding protein domain and the N-terminal of this product corresponds (100 % similarity) with the Oviposition Stimulating Protein (OSP) [53] (Additional file 6: Figure S3). BLASTp alignments showed significant homology between OSP and described proteins of the take out/Juvenile hormone binding proteins (TO/JHBP). Phylogenetic analysis showed OSP clustering with a large group of highly diversified takeout (TO) proteins including other known insect SFPs containing TO/JHBP domains. However, these SFPs do not form a monophyletic group (Fig. 3). Approximately 26 % (*n* = 46/176) of the secreted transcripts did not show any significant similarities with annotated domains, suggesting that the LHT secretome is enriched in novel SFPs.

Because previous studies in spermatophore producing insects have shown that there is some functional conservation of SFPs across distantly related taxa [14, 32, 40, 42], we cross-referenced the list of 176 predicted secreted proteins (Additional file 7: Table S4) with a database containing putative SFPs previously identified in other insect species (see Methods). Using reciprocal BLAST we were able to find putative orthologous for (36) proteins described in this study, but only 28 of them had a bit score >100. As expected, most orthologous (~43 %, *n* = 12) were found in other orthopterans and only a few were found in more distantly related taxa (n_*Heliconious*_ = 5, n_*Tribolium*_ = 4, n_*Aedes*_ = 7; Table1).

**Fig. 4:**
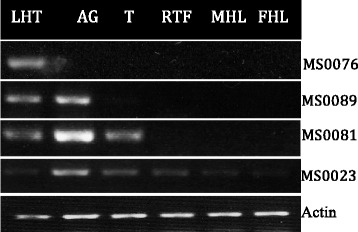
Representative example of the qualitative reverse transcription (RT-PCR) expression assays*.* Accessory gland (AG), testes (T), Reproductive female tissues (spermatheca + oviduct) (RFT), male head and leg (MHL) and female head and leg (FHL)

Although previous studies suggest that not all SFP encoding genes are exclusively expressed in male reproductive tissues [28, 29] male-biased expression is likely to be expected. Thus, we looked at the expression patterns in the group of putative SFPs that had a canonical signal peptide and/or showed significant orthology with previously reported reproductive proteins (see Fig. 4, Table 1). Our results showed that approximately 30 % (19/64) showed male expression bias (*i.e*. expressed in males but not in females), of which 12 corresponded to proteolysis regulators, two are lipases, three have unknown functions, and one encodes OSP.

**Table 1:** Putative SFPs identified in the LHT of *Melanoplus sanguinipes*

Contig	Expression patterns	Functional summary	Reads	EmPAI	Genome matched (Reference)	BLASTP description	Orthology			Signal secretion
							E-value	Hit Rank	Bit score	
*Proteolysis regulators*										
MS0199	a	Dipeptidase	58	6.07	*A. fasciatus* (2)	EG018903.1 Af_ARG_10E12_sp6 Accessory gland	6.00E-63	2	208	SP
MS1698	a	Serine protease inhibitor	60	1.06	*H. melpomne* (2)	HM023856.2| SFP HACP059	4.00E-45	1	139	SP
MS3398	a	Serpin b6	21	2.15	*H. melpomene* (2)	HM023856.2 SFP HACP059	9.00E-23	4	134	SP
MS1783	a	Angiotensin-converting enzyme	93	0.96	*L. migratoria* (3)	AY487174.1| Angiotensin converting enzyme	0	1	803	SP
MS0381	a	Metallo-endopeptidase (nepriyisin)	36	0.98	*G. firmus* (1)	EB409838.1 Gf_AcGl1_07A05_SP6 AcGl1	2.00E-34	1	127	SP
MS0081	a	Serine protease	481	6.21	*A. fasciatus* (1)	GQ911573.1 ejaculate serine protease (ejac-sp)	5.00E-80	1	168	NC
MS0089	a	Serine protease	481	28.8	*H. melpomene* (2)	HM023850.1 SFP HACP049	7.00E-61	1	119	NC
MS3857	a	Serine carboxypeptidase	6	1.5	*Ae. Aegypti* (6)	XM_001660689.1 Zinc carboxypeptidase	6.00E-48	1	116	NC
MS2174	b	Serine protease	29	2.17	*H. melpomene* (2)	HM023834.1 SFP HACP027	3.00E-36	1	148	NC
MS1693	a	Serine protease	175	0.29	*H. melpomene* (2)	HM023850.1 SFP	2.00E-21	1	40.5	SP
MS3819	b	Transmembrane protease serine	6	0.6	*G. firmus* (1)	DQ520137.1 Accessory gland protein (AG-0159F)	1.00E-10	4	46.4	SP
MS2043	a	Serine protease	8	0.75	*G. firmus* (1)	EB409712.1Gf_AcGl1_08D06_SP6	46.0E-10	4	45.5	SP
MS5974	b	Serine protease inhibitor	4	0.2	-	-	-	-	-	SP
MS0294	a	Serine carboxypeptidase	146	9.86	-	-	-	-	-	SP
MS6504	a	Serine carboxypeptidase	8	1.26	-	-	-	-	-	SP
*Environmental stress proteins*										
MS0126	c	Heat shock protein	84	0.79	*T. casteanum* (5)	XM_965476.2 70kD protein cognate (LOC659147)	1.00E-155	1	538	SP
MS0131	b	Heat shock protein	17	1.59	*T. casteanum* (5)	XM_961518.2. 70kD protein cognate 70, LOC657158	0	1	1216	-
*Iron storage proteins*										
MS1628	b	Transferrin	33	4.26	*G. firmus* (1)	EB409584.1 Gf_AcGl1_03G10_SP6 AcGl1	2.00E-69	1	256	SP
MS0380	b	Ferritin subunit	51	0.14	-	-	-	-	-	SP
*Oxidative stress proteins*										
MS1782	b	Catalase	4	1	*Ae. Aegypti* (6)	XM_001663550.1 Aedes aegypti catalase partial	4.00E-115	1	290	SP
MS0023	b	Aldo-keto reductase	323	2.56	*H. erato* (2)	HM023814.1 Seminal fluid protein HACP058	2.00E-57	1	102	NC
MS2301	c	Phenoloxidase	26	1.69	*T. casteanum* (5)	NM_001039433.1| Tribolium castaneum pro-phenol oxidase subun…	0	1	286	NC
MS0678	c	Glutathione s-transferase	12	2.88	*Ae. Aegypti* (6)	XM_001661819.1 glutathione s-transferase	4.00E-55	1	203	-
MS0031	b	Endoplasmin	142	0.17	-	-	-	-	-	SP
MS4425	b	Aldehyde oxidase	7	0.3	-	-	-	-	-	SP
					*Lipases*					
MS5352	c	Lipase	10	4.85	*Ae. Aegypti* (6)	XM_001651399.1| vitellogenin partial	1.00E-24	1	109	SP
MS1414	a	Lipase	19	1.53	-	-	-	-	-	SP
MS0358	a	Lipase	119	21.37	-	-	-	-	-	SP
*Cell adhesion protein, membrane-bound and structural proteins*										
MS2129	b	Moesin ezrin radixin	42	3.91	*Ae. Aegypti* (6)	XM_001652925. Moesin/ezrin/radixin	0	1	903	
MS0111	b	Actin	446	2.56	*G. firmus* (1)	EB409566 Gf_AcGl1_03F03_SP6 5	1.00E-92	1	333	NC
MS0014	b	Annexin isoform a	85	0.11	*T. casteanum* (5)	XM_962838.2 PREDICTED: similar to Annexin IX CG5730-PC	9.00E-147	1	515	-
MS0238	b	Elongation factor 1-alpha	238	1.67	*A. fasciatus* (2)	EG018577.1 Af_ARG_09B03_sp6 Accessory gland	3.00E-127	1	449	-
MS0234	b	Elongation factor 2	864	0.46	*G. firmus* (1)	EB409600.1 Gf_AcGl1_01A02_SP6 AcGl1	2.00E-128	1	453	-
*Others*										
MS0219	b	Calreticulin	343	0.37	-	-	-	-	-	SP
MS0105	b	Midline fasciclin	287	4.08	*A. fasciatus* (2)	EG018790.1 Af_ARG_02F01_sp6 Accessory gland	2.00 E-75	1	169	SP
MS0076	a	Hypothetical EAI_15996	6173	73.01	*G. firmus* (1)	EB409920.1 Gf_AcGl1_06A06_SP6	2.00E-47	1	184	SP
MS0157	b	Nucleoside diphosphate kinase	154	1.43	*Ae. Aegypti* (6)	XM_001662462.1Aedes aegypti nucleoside-diphosphate kinase	3.00E-87	1	310	SP
MS1368	b	Protein 5nuc-like	99	0.66	*G. firmus* (1)	EB409815.1 Gf_AcGl1_09F10_SP6	3.00E-30	1	110	SP
MS0047	b	Peptidyl-prolyl cis-trans isomerase 5	106	4.97	*G. firmus* (1)	EU669817.1 Peptidyl-prolyl isomerase-1	3.00E-45	2	174	SP
MS1669	c	Aminopeptidase -like	12	0.18	*Ae. Aegypti (*6)	XM_001656177.1 Aedes aegypti leucyl amino peptidase partial	2.00E-79	1	142	-
MS1026	b	Peptidoglycan recognition protein	23	2.22	-	-	-	-	-	SP
MS2092	b	Hypoxia up-regulated protein	12	0.73	-	-	-	-	-	SP
MS7155	a	Juvenile hormone-inducible	7	0.12	-	-	-	-	-	SP
MS0013	a	Oviposition Stimulating Protein (OSP)	28685	151323.3	-	-	-	-	-	SP
MS0682	b	Proactivator polypeptide	209	0.08	-	-	-	-	-	SP
MS0237	c	Carbonic anhydrase 2-like	18	1.56	-	-	-	-	-	SP
MS0221	c	Neutral alpha-glucosidase ab-like	24	0.45	-	-	-	-	-	SP
MS0644	b	Profilin	149	0.54	-	-	-	-	-	SP
MS2078	c	Plasma alpha-l-fucosidase	21	1.3	-	-	-	-	-	SP
MS0168	c	Esterase fe4-like	32	1.81	-	-	-	-	-	SP
MS0673		AaeL_AAEL001498	24	0.55						SP
MS3488	b	Hemomucin	10	1.07	-	-	-	-	-	SP
MS0097	b	Pdgf- and vegf-related factor 1-like precursor	6	0.26	-	-	-	-	-	SP
MS1006	b	Imaginal disc growth factor 4	75	4.26	-	-	-	-	-	SP
MS1117	c	Calumenin	19	0.23	-	-	-	-	-	SP
*Unknown*										
MS0327	c	Unknown	164	1.54	-	-	-	-	-	SP
MS1333	a	Unknown	55	3.39	-	-	-	-	-	SP
MS0039	a	Unknown	681	1.43	-	-	-	-	-	SP
MS2957	b	Unknown	13	0.18	-	-	-	-	-	SP
MS0784	b	Unknown	7	3.09	-	-	-	-	-	SP
MS0278	c	Unknown	375	0.51	-	-	-	-	-	SP
MS0281	c	Unknown	167	0.44	-	-	-	-	-	SP
MS5071	b	Unknown	2	1.92	-	-	-	-	-	SP
MS1809	c	Unknown	33	1.71	-	-	-	-	-	SP

References ((1) Andres et al. [14, 40]; (2) Walters and Harrison. [32] (3) Macours et al. [107]; (4) Findlay et al. [29]; (5) Xu et al. [24]; (6) Sirot et al. [58]). *E*xpression patterns using qualitative RT-PCR [(a) Male expression exclusively; (b) Expression in both sexes and (c) Not amplified. Signal secretion [Signal peptide (SP), Non-canonical secretion (NP)

## Discussion

Male’s seminal fluid contains peptides that modify almost all aspects of female reproductive physiology and behavior. Here, we have capitalized on genomic and proteomic techniques to characterize the SFPs secreted by the LHT gland of *M. sanguinipes* [14, 19, 24, 28, 31, 35, 65]. Our analyses revealed that the secretome of the LHT is complex.

A potential approach to identify *bona fide* SFPs is to consider only those proteins that either showed orthology with SFPs described in other insects and/or that have canonical signal peptide. Using exclusively these overstrict criteria, we have been able to identify as many as 64 different putative SFPs (Table 1). However, this number does not include rapidly evolving SFPs and genes with unknown functions with non-canonical secretion signals, which may represent a significant fraction of the ejaculate [33, 40, 77]. Assuming that our non-canonical secretion analyses generated only a few (~5 %) false positives, and that all secreted proteins are part of the seminal fluid, the number of identified putative SFPs raises to 176 (Fig. 2). This latter number is likely to be a better estimate of total number of SFPs secreted by the LHT, which seems to be higher than that described in other insects using similar methods (mean *N*_Secreted-SFPs_: 38; range: 13-138 (reviewed in [7, 24, 77])). Although this difference might just reflect the variation in experimental design (or in the interpretation of the results) among published studies is it also possible that the LHT secretome is particularly heterogeneous. Interestingly, a large number of putative SFPs have also been described in *Gryllus* and *Allonemobius* crickets, suggesting that Orthopterans ejaculates may be especially complex [14, 40, 42, 46, 77].

The characterization of the LHT secretome revealed several important patterns. First, the presence of proteins that lack canonical signal peptides in accessory gland proteomes is indeed common [19, 35]. In the LHT of *M. sanguinipes* only ~28 % (49/176) of the proteins predicted to be secreted contain a signal peptide. This result highlights the importance of nonstandard routes in the secretion of SFPs. Second, the products of a few highly expressed genes dominate the secretion of accessory glands. Previous studies in crickets have suggested that the highly expressed genes of the accessory glands encode structural (*i.e.* spermatophore building) proteins [40, 42]. However, this is not necessarily the case in the LHT of the migratory grasshopper. The most abundant product of the LHT was a protein identified as OSP, known to stimulate oviposition in migratory grasshopper females [53]. Third, accessory gland transcriptomes are enriched in novel transcripts. 26 % (46/176) predicted secreted SFPs identified in the proteomics experiment lack annotated domains, suggesting that this gland is enriched with highly abundant novel seminal fluid proteins coding genes whose function in reproductive behavior has yet to be evaluated. Many of the other male-expressed and LHT secreted proteins described in this study belong to the same functional classes as previously described SFPs including proteins involved in processing and degradation of proteins [26, 27, 36, 78], odorant/hormone binding-like proteins [31, 60, 79], immune and stress responses [19, 34, 35], and metabolic pathways and/or structural proteins with known functions related to reproduction. In what follows we discuss the potential roles of the LHT-SFPs that were identified using both the proteomics and genomics experiments*.*

### Take-out (TO) and Juvenile hormone binding proteins (JHBPs)

*TO/JHBPs* are small proteins (~240 amino acids) found exclusively in insects, and are involved in the transportation of hydrophobic ligands [80]. Putative SFPs containing TO/JBHP domains have been previously described in the male accessory glands of *Heliconious* butterflies, the Mediterranean fruit fly (*Ceratitis capitata*) and the flour beetle, *Tribolium castaneum* [19, 24, 32], and may be involved in the transfer of small hydrophobic molecules during copulation.

Homology based analyses revealed that OSP [53], the most abundant protein in the LHT, belongs to this group. Juvenile hormone (JH) is known to stimulate oogenesis [81]. Thus, there is the possibility that OSP acts as a carrier for JH. However, our distance-based phylogenetic tree clustered OSP with the highly diversified family of *TO* proteins instead of with known JH-binding proteins (Fig. 3). This result suggests that OSP is not likely to be involved in JH transportation. In fact, extensive biochemical and molecular functional characterization of this protein have revealed that it has little affinity for JH [82].

### SFPs involved in the synthesis of prostaglandins

Prostaglandins (PGs), prostaglandin-precursors and prostaglandin-synthesizing enzymes have been found in the testes, and the accessory glands of different insect’s groups [83–87]. Our bioinformatics analyses revealed the presence of prostaglandin F synthase (PGF_2α_ MS4100) and prostaglandin E synthase 2 (PGE_2_, MS5577) in the LHT. In at least two crickets species (*Acheta domesticus* and *Teleogryllus commodus)* short-term oviposition is stimulated by the post-copulatory synthesis of prostaglandins in the female’s reproductive tract [84, 85, 88]*.* Thus, LHT-PG synthetases may play a similar role in *M. sanguinipes*. However, this function is not necessarily conserved across orthopterans and prostaglandins do not appear to affect egg-laying in the migratory locust, *Locusta migratoria* [86].

The synthesis of prostaglandins also involves the oxidation of lipids and the release of reactive oxygen species (ROS), which might cause DNA damage, membrane degradation and premature activation of the sperm. Therefore, antioxidant SFPs may be particularly important in those species in which prostaglandins are stored and/o synthesized in the sperm storage organs of the females [89–91]. Accordingly, in our study we have identified 16 putative SFPs with antioxidant properties, 5 of which are orthologous of SFPs found in other insect species [Glutathione S-transferase (GST) contigs (MS4391, MS0677 and MS678), catalase (CAT) contig (MS1782), phenoloxidase, contig (MS2301), aldo keto reductase contig (MS0023)] [19, 92].

Three different transcripts (MS358, MS1414 and MS5352) that have predicted signal peptides and which expression is restricted to the LHT showed significant homology with different lipases found in the seminal fluid of other insects [41, 66, 84, 85]. Although an obvious function of these enzymes is to provide energy to sperm by the hydrolysis of triglycerides [34], lipases are also known regulators of the complexes that catalyze the conversion of arachidonic acid to prostaglandins inside the female’s reproductive tract [93]. Thus, it is possible that these LHT-SFPs may help to regulate the synthesis of prostaglandins in the spermatheca of the mated females.

### Proteolysis regulators

Proteolysis regulators are common key modulators of insects’ reproductive physiology [19, 24, 27, 35, 38, 46]. Our results showed that this functional class represents a significant fraction of the LHT secretome. In total, 23 putative SFPs correspond to proteases or their inhibitors. These include 12 serine proteases, 5 serine protease inhibitors (SERPINs), 4 carboxypeptidases, 2 neprylisins metalloendopeptidases, and 1 dipeptidase. Proteolysis regulators secreted by the LHT are highly conserved, and for all but one we have found orthologous proteins in distantly related species of insects. Among them, we have identified a gene (contig MS0081) that encodes a highly expressed trypsin-like serine protease, which is the putative orthologous protein of the ejaculate serine protease *ejac-sp* gene. Along with *OSP, EJAC-SP is* one of the most abundant proteins in the seminal fluid of the ground cricket *Allonemobious socius,* and is one of the few peptides known to mediate oviposition in orthopterans [46].

Zinc-dependent metallopeptidases, including neprilysins and angiotensin-like converting enzymes, are important proteolysis regulators. The predicted MS381product shows functional homology with different neprilysins, a family of proteins known to play important roles in both spermatogenesis and fertilization [94]. In insects, different isoforms of a highly conserved angiotensin-converting enzyme (ACE) are expressed in male accessory glands [24, 38, 95, 96]. The contig MS1783 shows significant orthology with *D. melanogaster* ANCE and *T. casteanum Lom-*ACE. While ANCE is involved in spermatid differentiation [95], the knockdown of *Lom-*ACE results in significant reduction in egg production by mated females and production of abnormal sperm [24]. It is possible that MS1783 plays similar functional roles in *M. sanguinipes*.

### Iron storage proteins

Two contigs (MS2388 and MS1628) correspond to a single predicted transferrin. Transferrins are iron binding proteins that have been reported in the male reproductive tract of blood-feeding insects [65, 80], but also in the accessory glands of the field cricket *T. oceanicus* [25]. Insect transferrins are multifunctional proteins [97, 98]. While in blood-feeding insects seminal transferrins may be related to blood utilization, in other insects groups such as orthopterans they may contribute to vitellogenesis, to immune functions, and /or to prevent oxidative stress [97]. In vertebrates, quantitative variation in transferrin in the seminal plasma correlates with sperm numbers, sperm motility, and male fertility [99–101], suggesting that transferrins may also play a role in sperm capacitation.

Secreted ferritins are known to be present in the hemolymph, the gut, and the ovaries of insects [98, 102]. These proteins have antioxidant activity [103] and are involved in innate immune responses [104, 105] and iron homeostasis [106], including iron store in eggs [102]. However, to the best of our knowledge they have not yet been reported in the seminal fluid of insects. Our proteomics analyses showed presence of a secreted ferritin homolog (contig MS0381) in the LHT. This result strongly suggests that ferritins are present in the seminal fluid of the migratory grasshopper. Though the function of ferritin-like proteins in the seminal fluid is not known, knockdown experiments have shown that the expression of these proteins affects oviposition and egg hatching rates in at least one species of tick [107].

### Metabolism-related proteins

In mammals, there is ample evidence that different (extra) cellular substrates and metabolic pathways are required to support the energetic requirements of sperm activation and fertilization. For example, while the acrosome reaction requires lactate or pyruvate for ATP production by oxidative phosphorylation, gamete fusion requires glucose to produce NADPH by the pentose phosphate pathway (reviewed in [108, 109]. In the LHT we have identified several secreted glycolitic enzymes, 5 of which [dehydrogenase, isocitrate dehydrogenase, α-enolase, gliceraldehyde-3-phosphate dehydrogenase, and nucleoside diphosphate kinase] have been found in the seminal fluid of the field cricket *Teleogryllus oceanicus* [77]. The roles and mode of action of most of these enzymatic SFPs in insects are not yet known. However, glucose dehydrogenases are required for sperm storage and utilization in *Drosophila* [110], and Neutral-α- glucosidase (contig MS0221) is required during sperm maturation in humans [111].

## Conclusions

In summary, this report is the first attempt at the identification of SFPs in the migratory grasshopper, *M. sanguinipes.* Using a combination of transcriptome and proteomic analyses we were able to identify 64 putative SFPs. Of special interest are relatively conserved genes, such as *EJAC-SP*, *ACE* and prostaglandin synthetases, products that are known to regulate female oviposition rate. Gene silencing has considerable promise for developing novel pest control techniques. However, functional characterization experiments in acridid grasshoppers and locusts are needed to assess if these SFP loci are useful targets for the implementation of this type of strategy.

## Availability of supporting data

DNA sequences of the seminal fluid proteins have been deposited in GenBank. (Accession numbers: KU218647-KU218708). Phylogenetic trees, and all other data are available through Dryad Digital Repository doi 10.5061/dryad.t80d3 (http://dx.doi.org/10.5061/dryad.t80d3.).

## Additional files

Additional file 1: Figure S1. Protein analysis of Long Hyaline Tubule (LHT) luminal secretions isolated from *M. sanguinipes* males on day 10 post-eclosion on 12 % SDS-PAGE. The entire gel lane was cut in 16 equal slices of approximately the same size (see Methods). (PDF 196 kb) Additional file 2: Table S1.
*Melanoplus sanguinipes* Seminal fluid proteins identified by mass-spectrometry. Predicted function based on the annotation and BLAST2GO. (XLSX 84 kb) Additional file 3: Table S2. Primers used in RT-PCR and PCR conditions. PCR was performed with the following thermal conditions 60 s at 95 °C, 50 s at 94 °C, 30 s at 62 °C, 3 cycles /°C declining over a range at 1 °C intervals of 30 s, 72 s at 60 °C, 50 s at 95 °C, 30 s at 60 °C, 6 cycles /°C declining over a range at 0.5 °C intervals of 30 s, 50 s at 95 °C, 30 s at 56 °C, 30 cycles of 30 s and 60 s at 72 °C. Relative expression was not quantified. (XLSX 49 kb) Additional file 4: Figure S2. Analyses of the LHT transcriptome from *M. sanguinipes* based on GO level II. A) Biological process. B) Component cellular. C) Molecular function. (PDF 452 kb) Additional file 5: Table S3. Transcriptome Annotation of the closest BLASTx hits (1e-5 cutoff). (XLSX 676 kb) Additional file 6: Figure S3. Protein sequence alignment of contig (MS0013) and N-terminal sequence protein of OSP obtained from Yi and Gillott. [53]. Signal peptide sequence is showed within the square. (PDF 73 kb) Additional file 7: Table S4. Predict secreted proteins based on the presence of a signal peptide (SP) or by non-classical secretory pathways (NC) (Scoring function > 0.5). *E*xpression patterns using qualitative RT-PCR to proteins with SP [(a) Male expression exclusively; (b) Expression in both sexes and (c) Not amplified. (XLSX 47 kb)

## Abbreviations

LHT: Long hyaline tubules
EJAC-SP: Ejaculate serine protease
ACE: Angiotensin-converting enzyme
SFPs: Seminal fluid proteins
AG: Accessory gland
OSP: Oviposition Stimulating Protein
NRC-Saskatoon: National Research Council Plant Biotechnology Institute Canada
GO: Gene Ontology
NCBI: Non-redundant databases of the National Center for Biotechnology information
ORFs: Open reading frames
LC-MS/MS: Liquid chromatography with tandem mass spectrometry
RBBHs: Reciprocal best Blast hits
JHBPs: Juvenile hormone binding proteins
T: Testes
MHL: Male head and leg
RTF: Female spermatheca and oviduct
FHL: Female head and leg
RT-PCR: Reverse transcription polymerase chain reaction
EST: Expressed Sequence Tags
EmPAI: Exponentially modified protein abundance values
JH: Juvenile hormone
TO: Take-out
PGF_2_: Prostaglandin F synthase
PGE_2_: Prostaglandin E synthase 2
PG: Prostaglandins
GST: Glutathione S-transferase Glutathione S-transferase
CAT: Catalase
ROS: Reactive oxygen species
SERPINs: Serine protease inhibitors
HSPs: Heat-shock proteins
