# Supplementary figures and images for: Combining RNA-seq and proteomic profiling to identify seminal fluid proteins in the migratory grasshopper Melanoplus sanguinipes (F)
Source: BMC Genomics. 2015 Dec 22;16:1096. doi: 10.1186/s12864-015-2327-1 (PMC4689059; doi:10.1186/s12864-015-2327-1)

**Figure S1.**

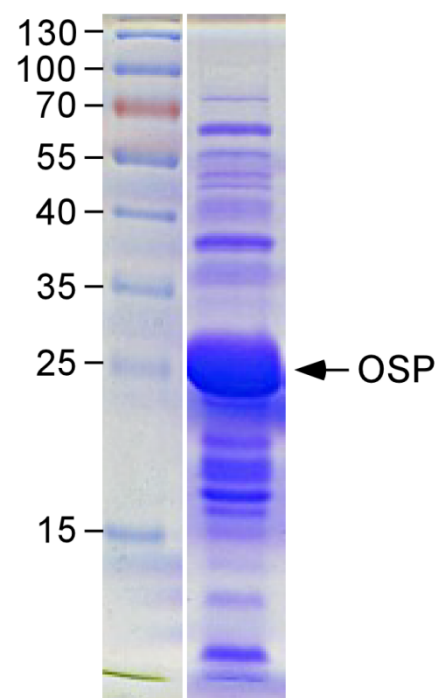

Supplement: Additional file 1: Figure S1. — Protein analysis of Long Hyaline Tubule (LHT) luminal secretions isolated from M. sanguinipes males on day 10 post-eclosion on 12 % SDS-PAGE. The entire gel lane was cut in 16 equal slices of approximately the same size (see Methods). (PDF 196 kb) [file 12864_2015_2327_MOESM1_ESM.pdf]

Figure S2

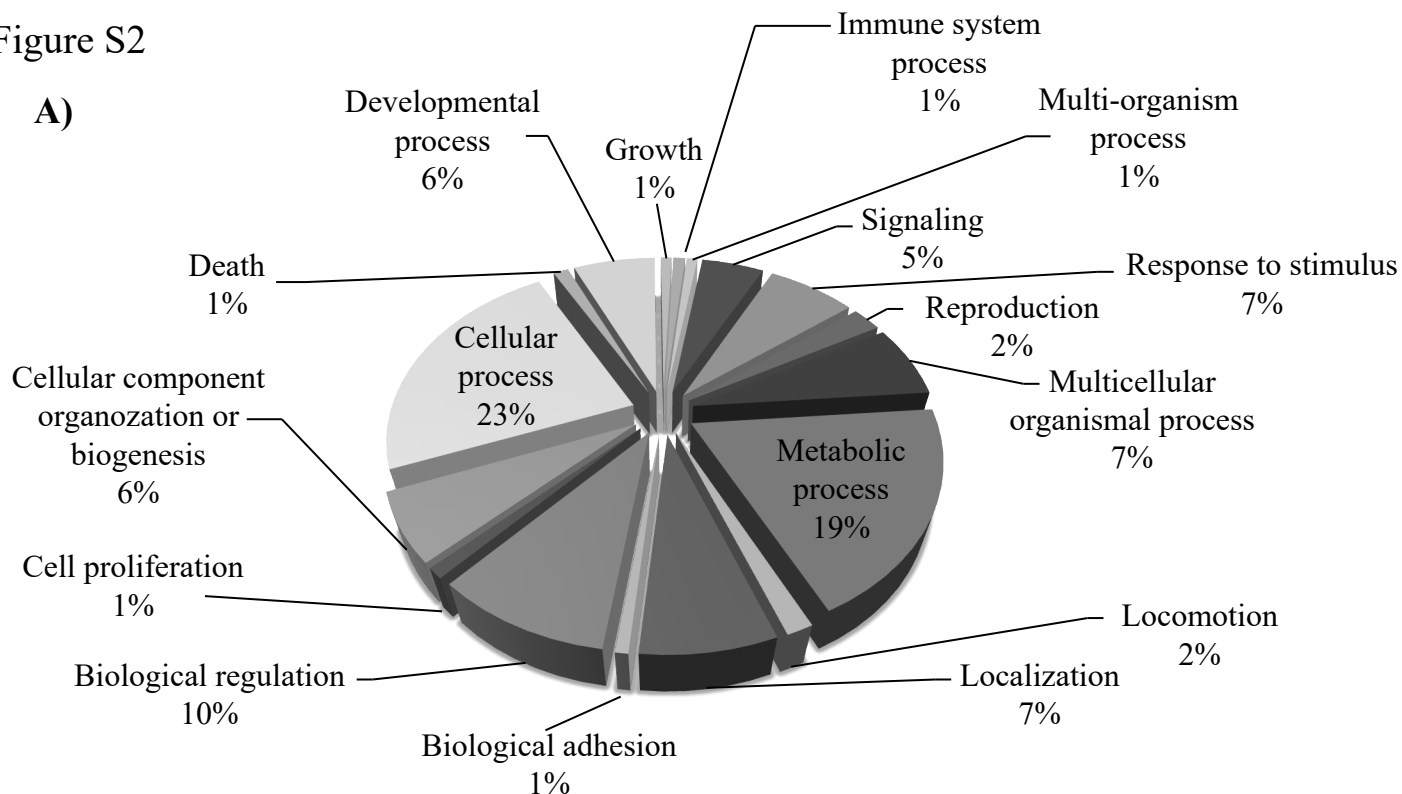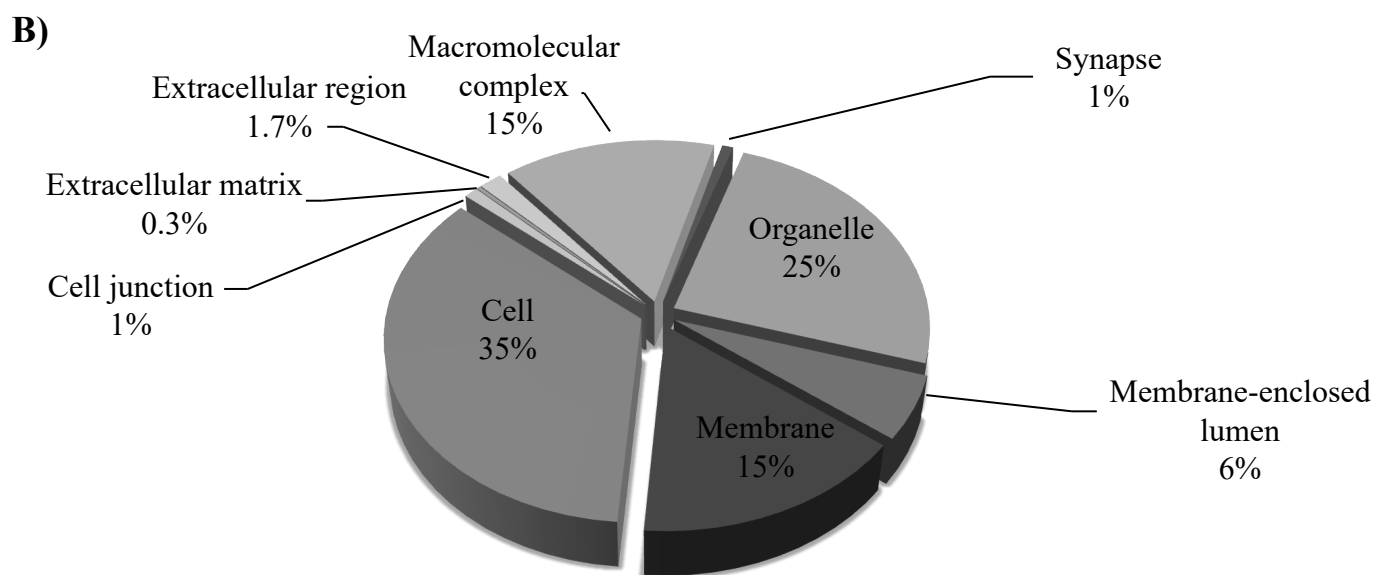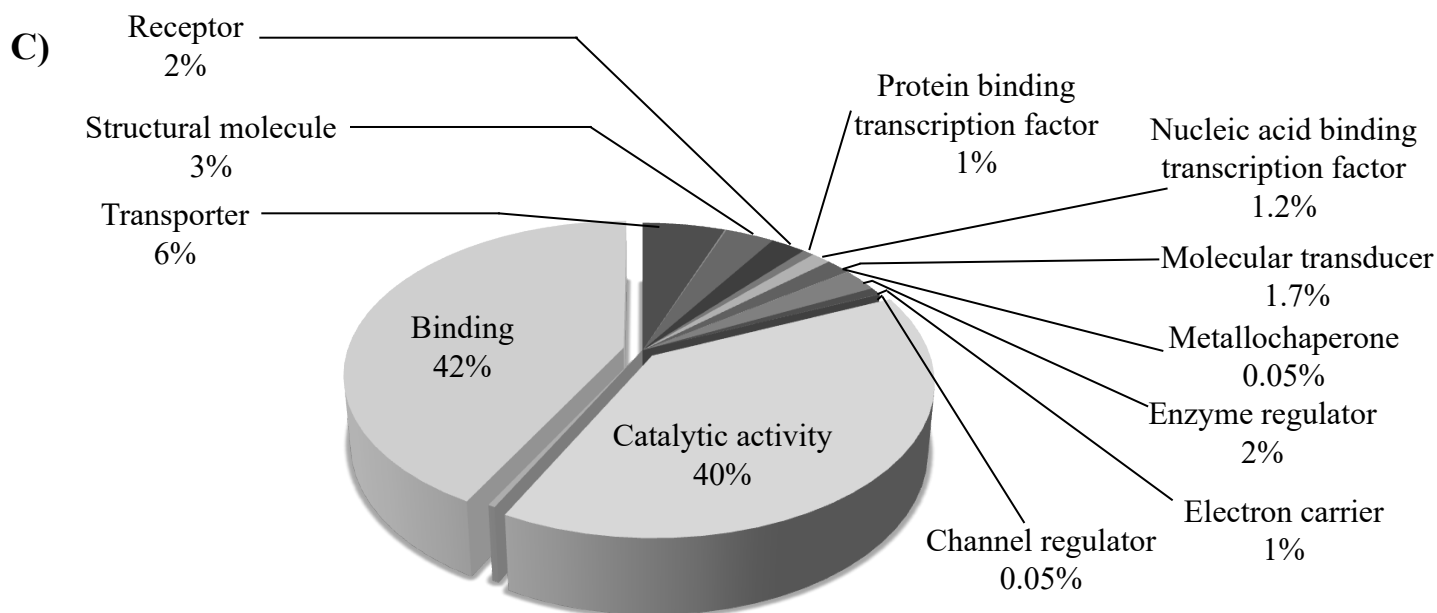

Supplement: Additional file 4: Figure S2. — Analyses of the LHT transcriptome from M. sanguinipes based on GO level II. A) Biological process. B) Component cellular. C) Molecular function. (PDF 452 kb) [file 12864_2015_2327_MOESM4_ESM.pdf]
