# Supplementary material for: Combining RNA-seq and proteomic profiling to identify seminal fluid proteins in the migratory grasshopper Melanoplus sanguinipes (F)
Source: BMC Genomics. 2015 Dec 22;16:1096. doi: 10.1186/s12864-015-2327-1 (PMC4689059; doi:10.1186/s12864-015-2327-1)
Supplement: Additional file 6: Figure S3. — Protein sequence alignment of contig (MS0013) and N-terminal sequence protein of OSP obtained from Yi and Gillott. [53]. Signal peptide sequence is showed within the square. (PDF 73 kb) [file 12864_2015_2327_MOESM6_ESM.pdf]

Figure S3. Protein sequence alignment of contig MS0013 and N-terminal sequence protein obtained from Yi and Gillott (1999). Signal peptide sequence is showed within the square.

```
                           10        20        30        40        50        60        70        80
                  ....|....|....|....|....|....|....|....|....|....|....|....|....|....|....|....|....|.
Contig MS0013     TGSGLRRSPLAPTTSATMSALQLCYLLAVAITSVTAALPSFVPACKKDDPKLDECFLNTINTIQPQMKKGIPELKIPTMEPLELSK
OSP N-Terminal    ------------------------------------ALPSFVPAWKKDDPKLDQQFF-----------------------------

                          110       120       130       140       150       160       170       180
                  ....|....|....|....|....|....|....|....|....|....|....|....|....|....|....|....|....|.
Contig MS0013     LENLKIHGAGDFKLINVHVDLKDNEFSGELEIPHLEFGADYNLALEVSNLTVKGKGDIKGTATDCKGNFVLKGELKERNGEKHFHF
OSP N-Terminal    --------------------------------------------------------------------------------------

                          210       220       230       240       250
                  ....|....|....|....|....|....|....|....|....|....|....|....
Contig MS0013     TLNGLIKGDVLMGDKHQHGAADMDLWLLLRPTIQDTIAEVAKDIVNGIAEVIVVDELFP
OSP N-Terminal    -----------------------------------------------------------
```
